# Andrographolide Sodium Bisulfate Prevents UV-Induced Skin Photoaging through Inhibiting Oxidative Stress and Inflammation

**DOI:** 10.1155/2016/3271451

**Published:** 2016-01-21

**Authors:** Janis Ya-Xian Zhan, Xiu-Fen Wang, Yu-Hong Liu, Zhen-Biao Zhang, Lan Wang, Jian-Nan Chen, Song Huang, Hui-Fang Zeng, Xiao-Ping Lai

**Affiliations:** ^1^School of Chinese Materia Medica, Guangzhou University of Chinese Medicine, Guangzhou 510006, China; ^2^The First Affiliated Hospital of Chinese Medicine, Guangzhou University of Chinese Medicine, Guangzhou 510405, China

## Abstract

Andrographolide sodium bisulfate (ASB), a water-soluble form made from andrographolide through sulfonating reaction, is an antioxidant and anti-inflammatory drug; however, the antiphotoaging effect of ASB has still not been revealed. Oxidative stress and inflammation are known to be responsible for ultraviolet (UV) irradiation induced skin damage and consequently premature aging. In this study, we aimed at examining the effect of ASB on UV-induced skin photoaging of mice by physiological and histological analysis of skin and examination of skin antioxidant enzymes and immunity analyses. Results showed that topical administration of ASB suppressed the UV-induced skin thickness, elasticity, wrinkles, and water content, while ASB, especially at dose of 3.6 mg/mouse, increased the skin collagen content by about 53.17%, decreased the epidermal thickness by about 41.38%, and prevented the UV-induced disruption of collagen fibers and elastic fibers. Furthermore, ASB decreased MDA level by about 40.21% and upregulated the activities of SOD and CAT and downregulated the production of IL-1*β*, IL-6, IL-10, and TNF-*α* in UV-irradiated mice. Our study confirmed the protective effect of ASB against UV-induced photoaging and initially indicated that this effect can be attributed to its antioxidant and anti-inflammatory activities* in vivo*, suggesting that ASB may be a potential antiphotoaging agent.

## 1. Introduction

**Figure 1:**
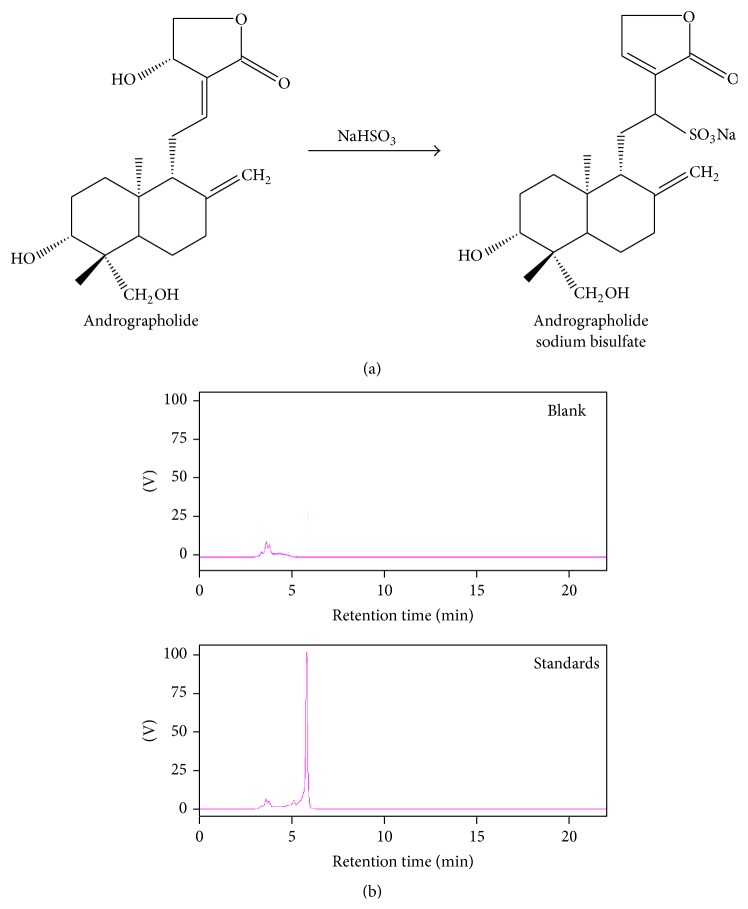
Andrographolide sodium bisulfate. (a) Andrographolide sodium bisulfate (ASB) is made from andrographolide through sulfonating reaction. (b) HPLC chromatogram of ASB.

*Andrographis paniculata* (Burm. f.) Nees (Acanthaceae) is a traditional herb that has been used in Taiwan, China, India, and other Southeast Asian countries for treating inflammation-related diseases such as rheumatoid arthritis and viral infections. Andrographolide is a labdane diterpenoid and is the well described pharmaceutical component of* A. paniculata*. Andrographolide is known to inhibit tumour metastasis, oxidative stress, and inflammatory responses and exhibit antimicrobial effects [1–3]. However, its efficacy is therapeutically decreased because of the poor water solubility and low oral bioavailability [4]. To improve the water solubility of andrographolide, a water-soluble form called andrographolide sodium bisulfate (ASB) is made from andrographolide through sulfonating reaction (shown in Figure 1). ASB has been reported to have similar pharmacological activities to andrographolide. Recent research has shown that ASB exerts its anti-inflammatory effect, which is associated with its action in suppressing inflammatory mediators and reducing oxidative stress [5, 6]. Currently, andrographolide and its derivatives have attracted wide attention for a skin care and an antiaging product due to their anti-inflammatory and antioxidative activities. However, the antiphotoaging effect of ASB has still not been revealed.

Skin aging is a complex, progressive process, which leads to functional and esthetic changes in the skin. Two main factors are responsible for this process, intrinsic as well as extrinsic processes [7]. Extrinsic process is due to environmental aggressors, also called as “photoaging” firstly coined in 1986. Photoaging is attributed to continuous, long-term exposure to ultraviolet (UV) radiation of approximately 280–400 nm, either natural or synthetic, on an intrinsically aged skin. Macroscopic differences for photoaged skin can be characterized by skin thickness, dryness, laxity, hyperpigmentation, leathery appearance, formation of coarse wrinkles, and loss of normal complexion [8–10]. In addition, the microscopic changes can be observed by epidermal hyperplasia, dermal elastosis, collagen degradation, and the presence of inflammatory infiltrates [9, 10]. UV-induced oxidative damage and inflammatory damage have been proved to play important roles in the pathogenesis of photoaging [11]. Generally, the ROS levels are regulated by the antioxidants such as superoxide dismutase (SOD), catalase (CAT), and glutathione peroxidase (GSH-Px). But if the balance of this oxidation/antioxidant system is broken by UV radiation, oxidative stress will occur in the deeper layers of the skin. Oxidative stress causes oxidative damage to cellular components in the skin, resulting in photodamage, photoaging, and photocarcinogenesis [12, 13]. The nuclear factor kappa B (NF-*κ*B) signaling pathway mediated by ROS is an important cell signaling pathway in the process of skin inflammation and aging. Excessive ROS induced by UV radiation can activate IkB kinase (IKK) which leads to IKB phosphorylation and promote the activation of NF-*κ*B. This activation can produce a variety of proinflammatory cytokines such as interleukin-10 (IL-10), IL-6, IL-1*β*, and tumor necrosis factor-*α* (TNF-*α*) and promote cell necrosis and apoptosis. It also can increase matrix metalloproteinases (MMPs) expression, degrade the extracellular matrix components, and cause skin relaxation, wrinkles, and erythema, thus leading to photoaging [14, 15].

In recent years, many compounds which possess antioxidant and anti-inflammatory properties have created considerable interest as protective agents for reducing UV-induced skin damage [11, 13, 16]. In this study, our aim was to evaluate the protective effect of ASB on mice skin photoaging induced by UV irradiation, particularly its antioxidant and anti-inflammatory properties.

## 2. Materials and Methods

### 2.1. Animals

Female KM mice (6 weeks old) were obtained from the Experimental Animal Centre of Guangzhou University of Chinese Medicine (GZUCM) in China. They were fed on standard laboratory diet and water* ad libitum* and housed in a controlled room of temperature at 23 ± 2°C, humidity at 50%  ±  10%, and light at 12 h light/dark cycle. All experimental protocols were approved by the Committee for Animal Care and Use at GZUCM (Approval number SCXK (Guangzhou)-2008–0020).

### 2.2. Chemicals

ASB was generously provided by Shanghai Haling biotechnology Ltd., Co., Shanghai, China. Its purity (>98%) was determined by high performance liquid chromatography (HPLC). The structure of ASB is shown in Figure 1(a). The UV-visible absorption spectrum of ASB dissolved in water is shown in Figure 1(b). Commercial kits for SOD and CAT and malondialdehyde (MDA) and the mouse hydroxyproline (Hyp) were purchased from Wuhan Cusabio Biotechnology Co., Ltd. (Wuhan, China). ELISA kits for TNF-*α*, IL-6, IL-1*β*, IL-10, and MMP-1 and MMP-3 were purchased from eBioscience, Inc. (San Diego, CA, USA). All other chemicals and reagents were of analytical grade.

### 2.3. Preparation of Photoaged Mouse Model

The irradiation procedure was described previously by Zhan et al. [17]. Briefly, mice were irradiated five times a week (except Monday and Thursday) for 10 weeks with UV irradiation device that includes seven UVB lamps surrounding three UVA lamps (Waldmann UV800, Germany). The irradiation intensity 30 cm from the light source was 1.0 mW/cm^2^. The minimal erythemal dose (MED) was preliminarily measured with a Waldmann UV meter (Waldmann Lichttechnik GmbH, Germany). The initial dose of UV was set at 70 mJ/cm^2^ assembled 1 MED for the first week. The irradiation dose was increase by 1 MED from 1 MED up to 4 MED and then kept at 4 MED for the remaining period of exposure [13].

After acclimatization for 1 week, the dorsal skin of mice (2.5 × 3 cm^2^), except those in the normal control group, were shaved with a safety razor and this operation was repeated before the UV periods. Female KM mice were randomly sorted into seven groups of eight mice each: (1) normal control (NC) group: mice were neither shaved nor treated and UV-irradiated; (2) sham control (SC) group: mice were only shaved but not provided with untreated sample and not irradiated with UV irradiation; (3) model control (MC) group: mice were provided with untreated sample and subjected to gradient-UV irradiation; (4) vehicle control (VC) group: mice were administrated topically with vehicle (water); and (5) ASB groups: mice were administrated topically with ASB (0.4, 1.2, and 3.6 mg/mouse) 2 h before UV irradiation. The mice were sacrificed by cervical dislocation under anesthesia at the end of the experiment. The appearance of the dorsal skin was measured by photographing each mouse using a digital camera. After that, the dorsal skins were collected. One section of these samples was harvested freshly and fixed in 10% neutral buffered formalin for histological examination. The other was quick-frozen and stored at −80°C for anti-inflammatory, antioxidant activities and protein measurements.

### 2.4. Physiological Analysis of the Skin Surface

To evaluate skin surface physiology, we measured the skin thickness, elasticity, wrinkles, and water content. In this protocol, the skin thickness was estimated weekly by a Quick Mini Caliper (Mitutoyo Co., Kanagawa, Japan). The skin elasticity was quantified by pinch test weekly in accordance with the modified protocol described by Tsukahara et al. [18]. Skin wrinkles began to be observed macroscopically in the dorsal region (site of wrinkle formation) after the initiation of UV irradiation. The skin macroscopic visual score was measured according to a grading scale based on the experimental model proposed by Bissett et al. [19]. The moisture of the skin was measured by drying the samples in an oven at 105°C for 4 h, as described by GB/T5009.3-2010, China [20].

### 2.5. Histological Examination

After being cleared in Histoclear (ASONE, Tokyo, Japan), the skin samples were stained by haematoxylin-eosin (H&E) and elastic Gomori's aldehyde fuchsin method to estimate skin structure alteration and elastosis as previously described [15, 21]. To quantify epidermal hyperplasia following UV exposure, 10 randomly selected cross sections per slide were photographed (magnification, 200x) by an optical microscope (Leica DMLB). Histological changes of each section were observed with the image analysis program Image J 1.36 (Wayne Rasband, National Institutes of Health, Bethesda, MD) [22].

### 2.6. Measurement of MMP-1 and MMP-3 Expression

Proteins are crucial factors for enriching target protein expression in the induction medium. To determine the amount of secreted MMP-1 and MMP-3 in the mouse skin, the skin tissue homogenate in PBS (v : v, 1 : 9) was centrifuged at 3000 ×g for 20 min at 4°C, and then the MMP-1 and MMP-3 in the supernatant were measured by ELISA kits. The assay procedure was performed manually and the absorption was measured at 450 nm.

### 2.7. Quantification of Collagen Hydroxyproline

Hydroxyproline (Hyp) is a major component of the protein collagen, which plays a key role for collagen stability. The concentration of hydroxyproline has been historically used as an estimate of collagen content [23]. Total Hyp content in the skin tissue was analyzed by ELISA kit according to the manufacturer's instructions.

### 2.8. Enzyme and Immunity Analyses

The SOD, CAT, and TBARS levels in skin homogenate were determined using SOD, CAT, and MDA assay kits, respectively. The IL-10, IL-1*β*, IL-6, and TNF-*α* level in the skin cytosolic fraction were measured by ELISA kits. All of the biochemical assays were performed according to the manufacturer's protocols of corresponding diagnostic kits. Moreover, protein concentration was evaluated using the method of Lowry and others using bovine serum albumin as standard [24].

### 2.9. Measurement of Spleen Index and Thymus Index

At the end of the study, the mice were euthanized and relevant organs were dissected out and weighed immediately. The thymus and spleen index were calculated to the formulas described as follows [22]: organ index (%) = average organ weight (in milligrams)/average body weight (in grams).

### 2.10. Statistical Analysis

Data were expressed as mean ± SD. Multiple group comparisons were performed using one-way analysis of variance (ANOVA) followed by Dunnett's test in order to detect intergroup differences. A value of *p* < 0.05 was considered to be statistically significant. All analyses were performed using SPSS (version 17.0).

## 3. Results

### 3.1. Clinical Observation: Effects of ASB on the Thickness, Elasticity, Wrinkles, and Water Content of the Skin Induced by UV Irradiation

During the experimental period, the body weight of the mice was measured regularly. Mean body weights of SC group (no UV irradiation), MC group (only UV irradiation), VC group (UV irradiation and only vehicle treatment), and ASB groups were similarly increased throughout the period of study. There was no significant difference in the mean body weight among the groups (data not shown). Food consumption was also not different among these groups (data not shown). Therefore, it seems that body weight gain and food consumption were not affected by UV irradiation and ASB treatment.

**Figure 2:**
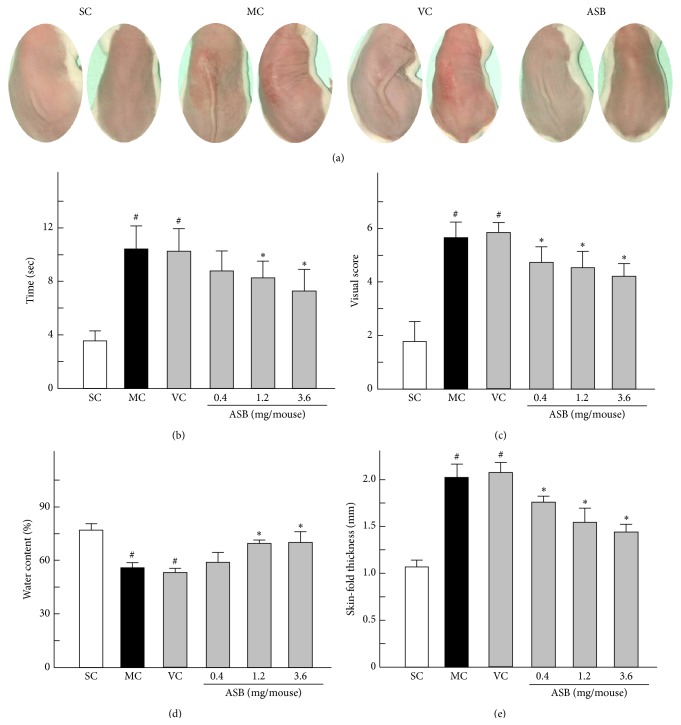
Evaluation of skin clinical observation at the end of experiment period. (a) (left) Pinch test was performed according to the method described by Tsukahara. (a) (right) Macroscopic changes of mice with different treatments were performed. (b) Recovery time in the pinch test of different groups was evaluated. (c) The trend of macroscopic changes reflected in the changes of visual scores was expressed according to the method described by Bissett et al. (d) Water contents of mice skin with different treatments were measured. (e) Skin-fold thickness of dorsal skin of different experiment groups was measured using a caliper. Data represents means ± SD (*n* = 8). ^#^ *p* < 0.05 compared with the SC group; ^*∗*^ *p* < 0.05 compared with the VC group. NC, normal control; SC, sham control; MC, model control; VC, vehicle control.

Skin elasticity reduced significantly during weeks 2 to 10 of UV irradiation. As shown in Figure 2(a) (left), the recovery time of the mice showed similarity between the MC group and VC group, but both values were rather greater than that in the SC group. The topical application of middle and high doses of ASB (1.2 and 3.6 mg/mouse) could reduce the recovery time markedly at week 10 compared to that of VC group mice (Figure 2(b)). The typical images in UV-induced macroscopic wrinkles formation in the dorsal region at tenth week were shown in Figure 2(a) (right). The SC group only showed age-related slight wrinkles. In contrast, after UV exposure, slight erythema and coarse wrinkles were formed in MC and VC groups. Wrinkle formation induced by UV exposure was inhibited by the topical application of ASB. As shown in Figure 2(c), 10 weeks of UV exposure induced a significant increase in the total score (5.8 versus 1.7 for the controls). Nevertheless, the visual scores of ASB groups were markedly decreased in a dose-dependent manner, when compared to VC group (4.5 and 4.1 versus 6.0 for the VC group).

On the other hand, the water content of the skin is greatly influenced by extrafibrillar matrix, which may be responsible for wrinkling and laxity of the skin accompanying the cutaneous aging [25, 26]. Therefore, water content in the skin is presumed to be a critical determinant in cutaneous aging. Excessive UV exposure of skin causes abnormal skin water loss. The water content of the skin in the SC mice was 75.74% (Figure 2(d)), which was notably decreased by 23.83% in the MC group and by 24.89% in the VC group. However, in ASB groups (1.2 and 3.6 mg/mouse), 12.60% and 15.15% of water were rescued, respectively (Figure 2(d)). This result showed that ASB prevented the loss of water contents and this effect is dose-dependent. Moreover, skin thickness increased significantly during weeks 3 to 10 of UV irradiation. The topical application of ASB, especially in the dose of 3.6 mg/mouse, significantly inhibited the increase in skin thickness induced by UV exposure at week 10 compared to the skin thickness of the mice in VC group (Figure 2(e)).

### 3.2. Histological Observation: Effects of ASB on the Thickness of the Epidermis and the Morphology in the Skin of UV-Irradiated Mice

**Figure 3:**
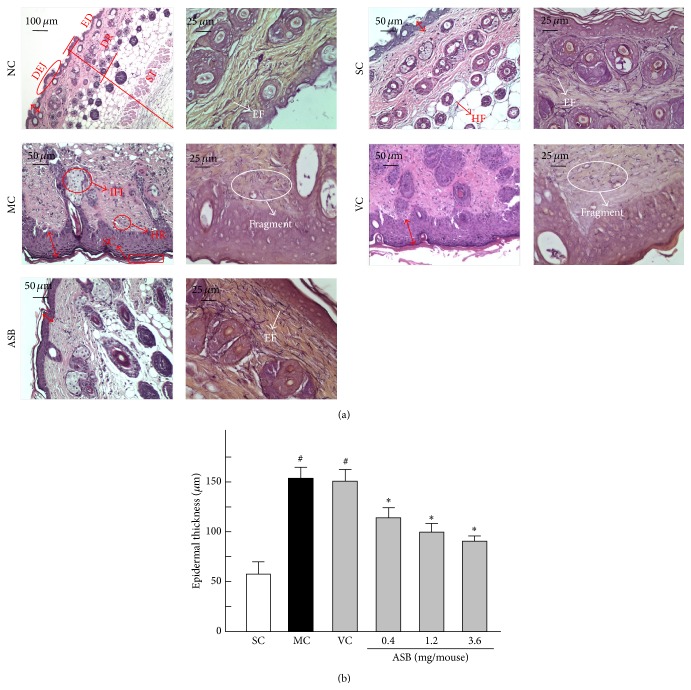
Evaluation of skin histological observation at the end of experiment period. (a) (left) H&E staining of mice skin for routine examination. (a) (right) Gomori's aldehyde fuchsin staining of mice skin. Epidermal thickness was shown via the double-headed red arrows. ED, epidermis; DR, dermis; ST, subcutaneous tissue; DEJ, dermal-epidermal junction; SC, stratum corneum; HF, hair follicle; HR, hemorrhage; IFI, inflammation infiltration; EF, elastic fibers. (b) Histogram presentation of the results of average epidermal thickness of each group at the end of experiment period. Data represents means ± SD (*n* = 8). ^#^ *p* < 0.05 compared with the SC group; ^*∗*^ *p* < 0.05 compared with the VC group. NC, normal control; SC, sham control; MC, model control; VC, vehicle control.

Histological analysis of the skin specimens were evaluated by routine H&E staining coupled with Gomori's aldehyde fuchsin staining. The thickness of the epidermis was measured on photomicrographs (Figure 3(a)). As shown in Figure 3(b), UV irradiation induced a 2.8-fold increase in the epidermal thickness relative to the normal group (NC) after ten weeks' irradiation. However, the topical application of ASB at a concentration of 0.4, 1.2, and 3.6 mg/mouse decreased the epidermal thickening caused by UV exposure by 25.50% and 30.93% and 41.38%, respectively.

After 10 weeks of UV irradiation of mice, we detected the characteristic histological features of the mice skin. Mice skin in NC group displayed a complete and clear structure (Figure 3(a)). SC group were not different from NC group, which explained that the shaving in this experiment had no effect on the histological features. The normal epidermis were covered by thin layer of stratum corneum, and the completed dermis showed orderly arranged collagen, abundant elastic fibers, and extrafibrillar matrix (NC and SC groups in Figure 3(a)). Histologically, UV irradiation caused a greater transformation of stratum corneum, as well as large quantities of abnormal, tangled, degraded, and nonfunctional fibers (Figure 3(a) MC). Moreover, dense inflammatory infiltrations as well as hemorrhage were evident in UV-exposed skin specimen (Figure 3(a) MC). Meanwhile, no significant difference in the skin features was observed between MC and VC mice skin (MC and VC groups in Figure 3(a)). However, the skin of mice in the ASB group showed well regular epidermis and dermis. Epidermal hyperplasia was significantly decreased and the epidermis had multiple layers of squamous cells, covered by thin layer of stratum corneum. In addition, in the dermal layer, clusters of sebaceous glands were attached to regularly distributed hair follicles; an ordered arrangement of abundant collagen bundles was displayed and the elastic fibers were normal in nature. Moreover, inflammatory infiltration and hemorrhage were absent in and underneath the fatty dermis (Figure 3(a), ASB). The results showed that the topical application of ASB produced significant protective effects on the UV-induced skin structure damage.

### 3.3. Skin Antioxidant Enzyme Activities

**Figure 4:**
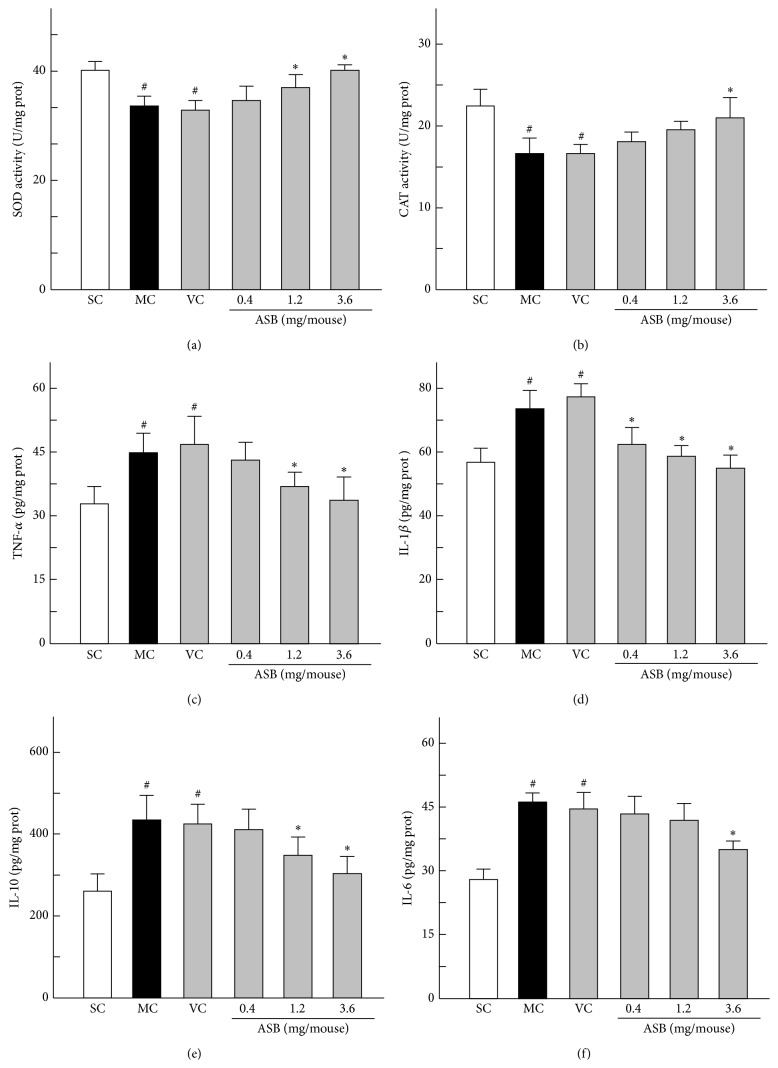
Effect of andrographolide sodium bisulfate (ASB) on the activities of antioxidant enzymes and the expression of inflammation cytokines in photoaged mice skin. Data represents means ± SD (*n* = 8). ^#^ *p* < 0.05 compared with the SC group; ^*∗*^ *p* < 0.05 compared with the VC group. NC, normal control; SC, sham control; MC, model control; VC, vehicle control.

Generally, skin antioxidant enzyme activities are recognized as important parameters to assess the antioxidant capacity in organisms. Among these enzymes, SOD and CAT are the major antioxidant enzymes protecting the epidermis [27, 28]. As shown in Figures 4(a) and 4(b), the SOD and CAT activities of MC group were significantly different from SC group, which showed that MC group was a successful photoaging model. ASB could protect the SOD and CAT against the damage in dose-dependent manners, as depicted by the significant increase of enzyme activities in ASB group mice. In particular the application of 3.6 mg/mouse of ABS could significantly increase the SOD by 15.43% and CAT by 23.74%, compared to that in the VC mice, respectively.

### 3.4. Cytokines Involved in Inflammation Processes in Dorsal Skin

The inflammatory cytokines (IL-1*β*, IL-6, IL-10, and TNF-*α*) levels were examined by ELISA in this study. UV exposure was shown to increase the contents of the inflammatory cytokines IL-1*β*, IL-6, IL-10, and TNF-*α* in the mice skin, while there was no significant difference in these cytokines levels between MC and VC groups (Figures 4(c), 4(d), 4(e), and 4(f)). However, the application of ASB obviously decreased these cytokines levels. Especially when treated with high dose of ASB (3.6 mg/mouse), IL-1*β* was increased by 21.57%, IL-6 was increased by 19.81%, IL-10 was increased by 26.30%, and TNF-*α* was increased by 18.75%. These results indicated that ASB could downregulate the excess skin levels of the inflammatory cytokines associated with UV exposure.

### 3.5. Skin MDA Production Evaluated by TBARS Assay

**Figure 5:**
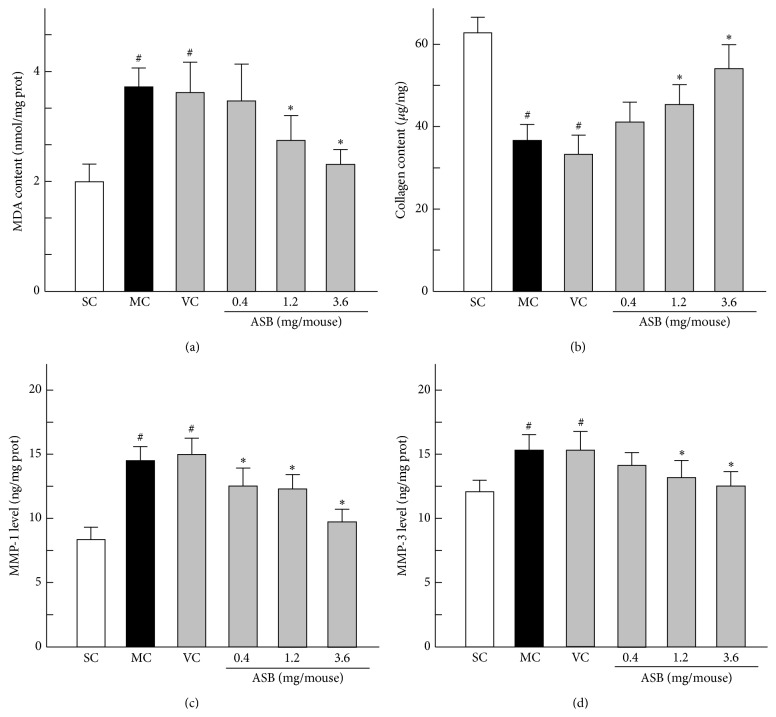
Effect of andrographolide sodium bisulfate (ASB) on the level of malondialdehyde (MDA) and collagen content and the expression of matrix metalloproteinase-1 (MMP-1) and MMP-3 in photoaged mice skin. Data represents means ± SD (*n* = 8). ^#^ *p* < 0.05 compared with the SC group; ^*∗*^ *p* < 0.05 compared with the VC group. NC, normal control; SC, sham control; MC, model control; VC, vehicle control.

The lipid peroxidation is considered as the main mechanism of cutaneous photoaging induced by UV. MDA is the lipid peroxidation product and is usually quantified to identify lipid peroxidation [29]. The levels of MDA equivalents of the mice skin were determined by TBARS assay. As shown in Figure 5(a), MDA contents were not significantly different between MC and VC groups. The MDA level in MC group was markedly increased by around 1-fold, compared to that in the SC group. However, the topical application of ASB inhibited the increased MDA content by UV-irradiation. MDA content was reduced by about 40.21% by treatment with 3.6 mg/mouse of ASB (*p* < 0.05, versus VC group).

### 3.6. Estimation of Collagen Content in Photoaged Mice

Collagen content can be determined through measuring the amount of Hyp. As shown in Figure 5(b), the MC and VC groups showed the similar decrease of collagen content when compared with the SC group, which explained that the vehicle solution had no effect on the collagen content. In the low dose of ASB group, the value of collagen production was close to that in VC group. However, the application of middle and high doses of ASB significantly increased the collagen content by 32.95% and 53.17%, respectively, as compared with the VC group. These results indicated that ASB prevented the UV-induced collagen damage.

### 3.7. Inhibition of UV-Induced MMPs' Contents by ASB

The induction of MMPs is suggested to be closely correlated with premature skin aging induced by UV in mice skin. To determine whether ASB inhibited UV-induced MMPs expression, MMP-1 and MMP-3 expressions were measured after 10 weeks' irradiation. As shown in Figures 5(c) and 5(d), the contents of MMP-1 and MMP-3 were not significantly different between MC and VC groups. In addition, UV exposure of skin markedly increased MMP-1 and MMP-3 contents by 78.61% and 28.36% compared to that in the SC group, respectively. However, ASB significantly prevented the increase of MMP-1 in a dose-dependent manner. Although the content of MMP-3 was similar between low doses of ASB group and VC group, the application of the dose of 1.2 and 3.6 mg/mouse ASB dramatically reduced MMP-3 contents in skin tissues (*p* < 0.05, versus VC group). These results indicated that ASB could inhibit the UV-induced increases of MMP-1 and MMP-3 expressions, consequently preventing the collagen degradation in the skin.

### 3.8. Thymus Index and Spleen Index

Bennett reported that UV exposure could affect the immune system in animals as well as in humans [30]. The spleen and thymus play important roles in regard to the immune system. Therefore, thymus and spleen indexes could be used as the indexes of immunity. As shown in Table 1, no significant difference was observed in thymus and spleen indexes either between NC and SC groups or between MC and VC groups; they also showed that repeated UV irradiation could significantly decrease thymus and spleen indexes of MC mice, which was 60.87% and 75.0% of SC mice, respectively (all *p* < 0.05 versus SC group). However, ASB could significantly increase the indexes of mice, compared with the MC group. ASB in different doses effectively increase the thymus index, although the value was lower than that in the NC group (Table 1). Similarly, spleen index in the mice of middle and high doses of ASB group significantly was increased when compared with that in the MC group, which had no significant difference with the NC mice (Table 1). These results suggested that administration of ASB could markedly enhance thymus index and spleen index, thereby protecting against UV-induced damage.

## 4. Discussion

UV irradiation is the main environmental hazard for causing skin photoaging, which includes the production of proinflammatory cytokines [31], suppression of the immune system [32], the generation of oxidative stress [15], and the expression of effector molecules like MMPs [33]. Many agents, possessing anti-inflammatory, immunomodulatory, and antioxidant properties, are gaining considerable attention for the prevention of UV-induced skin damage [11, 13, 16]. ASB is a kind of soluble derivative of andrographolide, which is the major bioactive compound isolated from* Andrographis paniculata*. Andrographolide and its derivatives are reported to have anti-inflammatory effect by suppressing inflammatory mediators and reducing oxidative stress [3–6]. Thus, in the present study, we administered ASB topically to the mice skin repeatedly exposed of UV irradiation and examined its effects on photoaging.

Consistent with the previous studies, our results showed that UV irradiation induced the increase of wrinkle formation and skin thickness and the reduction of skin elasticity [34]. On the one hand, the water content of the skin is mainly affected by ground substances, which may bring about wrinkling and laxity of the skin, even an exaggerated epidermal hyperplasia being associated with cutaneous aging [26]. It was found that excessive UV exposure of skin caused abnormal skin water loss. On the other hand, it is suggested that elastic accumulation and collagen degradation in the skin dermis were responsible for the wrinkled appearance and sagged skin [35]. Histopathologically, excessive UV exposure resulted in the tangled and broken collagen fibers and the decreased number of elastic fibers and disorganized elastic fibers. The histochemical assay also showed that collagen content was lower as a result of UV irradiation, as reported previously [36]. Moreover, it is known that UV irradiation can induce synthesis of various destructive enzymes specifically the matrix metalloproteinases (MMP-1, MMP-3) in dermal fibroblasts which is responsible for tangled and degraded collagen and elastic fibers, therefore leading to cutaneous photoaging [10, 37]. In this study, increased MMP-1 and MMP-3 expressions by UV showed consistency with previous investigations [38]. However, in comparison with the VC group, the ASB treated group had a better skin appearance, in which the topical application of ASB suppressed the wrinkle formation, sagged appearance, and skin thickness. Moreover, ASB (especially 1.2 and 3.6 mg/mouse) not only markedly prevented the loss of water contents induced by UV irradiation but also inhibited the degradation of collagen and repaired the integrity of collagen structure and elastic fibrous tissue. These data indicated that ASB could alleviate skin wrinkles and laxity, which resulted from its fixing collagen and elastic fibers and inhibiting the skin water loss. Moreover, the topical application of ASB suppressed the UV-induced increase of MMP-1 and MMP-3 expressions, which might lead to remodeling extracellular matrix structures in the tissue treated with ASB.

A growing body of evidence suggests that UV-generated ROS stimulates membrane dependent MAPK signaling pathways and the transcription factor activation protein 1 (AP-1) and hence leads to the upregulation of MMP production [11]. It has been reported that free radical scavengers and antioxidants such as genistein [39], EGCG [32], and delphinidin [40] could alleviate the UV-induced oxidative stress to protect skin from the injury. Normally, the skin possesses an endogenous antioxidant defense system to scavenge the formed ROS and deal with UV-induced oxidative stress. Our investigation showed that UV-induced oxidative stress came along with accelerating lipid peroxidation (LPO) and reducing the activity of SOD and CAT. However, topical application of ASB could significantly prevent the decrease in these enzymes activities and attenuated the augment of MDA levels, a reliable laboratory biomarker to evaluate the degree of LPO. These results indicated that the antiphotoaging property of ASB was due to its antioxidant role to suppress ROS formation, protect antioxidant enzyme system, and thus lead to the decreased MDA levels, which coincided with previous results [12, 13].

In addition to altering cellular redox equilibrium leading to ROS formation and membrane LPO, UV irradiation has also been shown to contribute to immunosuppression [32]. Cytokines and chemokines are very important in immunologic regulation in the human body and are associated with the induction of proliferation, differentiation, and cell death [41]. For example, interleukin-1*β* (IL-1*β*), IL-6, IL-10, and TNF-*α* are involved in inflammatory responses* in vivo*. Erythema, epidermal hyperplasia, and infiltration of inflammatory cells after exposure to UV are considered as inflammatory responses and play an important role in the development of skin photoaging which promotes the expression of MMPs and destroys the integrity of skin cellular and molecular components through proinflammatory cytokines [14]. Studies have suggested that UV-induced ROS production is involved in the activation of AP-1 and nuclear factor-*κ*B (NF-*κ*B) to lead to the production of cutaneous proinflammatory cytokines such as IL-1*β*, IL-6, and TNF-*α* [42]. In addition, studies manifested that the overexpression of the immunosuppressive cytokine IL-10 has been involved in the UV-induced skin inflammatory damage [43]. Our results showed that UV exposure induced erythema, edema, and epidermal hyperplasia of the mice skin, while upregulating the levels of IL-1*β*, IL-6, TNF-*α*, and IL-10 in mice skin significantly. However, the levels of these cytokines were markedly reduced when treated with ASB at a dose of 1.2 and 3.6 mg/mouse. Histological results also revealed that inflammatory cells gathering area was absent in the ASB group, which was proved by satiny skin shown in Figure 3. These results indicated that ASB could improve the skin immunity activities by suppressing the UV-induced production of inflammatory cytokine.

Moreover, the UV-induced suppressor T cells mediate their suppressive effects by releasing immune regulatory cytokines, particularly IL-10 [44]. Several different regulatory T cells have been considered as being involved in the various different models of UV-mediated tolerance [44]. It is clear that suppressor T cells reside in the lymphoid organs of UV-irradiated mice. The spleen and thymus are two lymphatic organs that play fundamental roles in the function of cellular and humoral immune system. Therefore, thymus and spleen indexes could be used as the indexes of immunity. Our study proved that UV irradiation suppressed the immune system, similar to a previous report, which showed that suberythemal doses of UV light could affect the immune system in mice as well as in humans [45]. However, the administration of ASB could markedly enhance the immunity activity of mice by increasing thymus and spleen indexes, thereby protecting against UV-induced damage.

## 5. Conclusion

In conclusion, the present study, for the first time, evaluated the protective effect of ASB on mice skin photoaging induced by UV irradiation. The mechanisms of this protection mainly involved downregulating MMP expressions, increasing antioxidant enzymes activities, and inhibiting the excessive cytokine production by UV irradiation. Therefore, the clinical application of ASB as a therapeutic and cosmetic product could be beneficial to protect against skin photoaging.

## Figures and Tables

**Table 1:** Effects of ASB on thymus index and spleen index on photoaged mice skin.

Group	Thymus index (mg/g)	Spleen index (mg/g)
NC	0.24 ± 0.01	0.35 ± 0.06
SC	0.23 ± 0.04	0.36 ± 0.05
MC	0.14 ± 0.02^#^	0.27 ± 0.04^#^
VC	0.16 ± 0.03^#^	0.27 ± 0.02^#^
ASB (0.4 mg/mouse)	0.19 ± 0.04^*∗*^	0.32 ± 0.08
ASB (1.2 mg/mouse)	0.18 ± 0.05^*∗*^	0.34 ± 0.04^*∗*^
ASB (3.6 mg/mouse)	0.20 ± 0.03^*∗*^	0.35 ± 0.04^*∗*^

Each value represents the mean ± SD of eight mice per group, ^#^ *p* < 0.05 compared with the SC group; ^*∗*^ *p* < 0.05 compared with the VC group.

## Abbreviations

ASB: Andrographolide sodium bisulfate
UV: Ultraviolet
SOD: Superoxide dismutase
CAT: Catalase
IL: Interleukin
TNF-*α*: Tumor necrosis factor-*α*
MMP: Matrix metalloproteinase
ROS: Reactive oxygen species
HPLC: High performance liquid chromatography
PBS: Phosphate buffered saline
MDA: Malondialdehyde
Hyp: Hydroxyproline
ELISA: Enzyme-linked immunosorbent assay
MED: Minimal erythemal dose
H&E: Haematoxylin-eosin
TBA: Thiobarbituric acid
TBARS: Thiobarbituric acid reactive substances
SD: Standard deviation
ANOVA: One-way analysis of variance
MAPK: Mitogen-activated protein kinase phosphorylation
AP-1: Activation protein 1
EGCG: Epigallocatechin gallate
LPO: Lipoperoxide
NF-kB: Nuclear factor kappa B.
